# Contrasting Waterlogging Response Strategies of Two Alpine Wetland Plants from Different Hydrological Niches: Insights from Root Traits, Rhizosphere Processes, and Transcriptomic Responses

**DOI:** 10.3390/plants15213290

**Published:** 2026-10-25

**Authors:** Xiaoyan Su, Changhui Li

**Affiliations:** 1 College of Forestry and Grassland, Qinghai University, Xining 810016, China; 2 Academy of Animal Husbandry and Veterinary Medicine, Qinghai University, Xining 810016, China

**Keywords:** waterlogging stress, hydrological niches, alpine wetland plants, root functional traits, rhizosphere functions, transcriptomics

## Abstract

Alpine wetland ecosystems on the Qinghai–Tibet Plateau are highly vulnerable to hydrological alterations, yet whether alpine wetland plants occupying different hydrological niches exhibit distinct adaptive strategies under waterlogging remains poorly understood. Here, two dominant alpine wetland species, *Kobresia tibetica* and *Blysmus sinocompressus*, were exposed to four waterlogging treatments: control (CK), slight waterlogging (SW), moderate waterlogging (MSW), and severe waterlogging (SSW). Root functional traits, rhizosphere soil enzyme activities, physiological responses, and root transcriptomic profiles were analyzed to elucidate species-specific responses to waterlogging stress. Slight waterlogging promoted root growth, whereas moderate and severe waterlogging inhibited root development and impaired physiological functions in both species. Root transcriptomic analysis under moderate waterlogging identified 123 and 2806 differentially expressed genes in *K. tibetica* and *B. sinocompressus*, respectively. These genes were mainly associated with redox regulation and stress responses in *K. tibetica*, whereas carbon metabolism, energy metabolism, and stress-related pathways were enriched in *B. sinocompressus*. Overall, the two alpine wetland species exhibited contrasting species-specific waterlogging response strategies, with *B. sinocompressus* showing greater capacity to maintain belowground functional stability under moderate and severe waterlogging. Quantitative reverse transcription polymerase chain reaction (qRT-PCR) validation further confirmed that waterlogging-responsive genes involved in anaerobic metabolism and antioxidant regulation showed species-specific expression patterns under severe waterlogging conditions.

## 1. Introduction

Alpine wetland ecosystems on the Qinghai–Tibet Plateau are highly sensitive to global climate change. Increasing temperatures, altered precipitation patterns, and permafrost degradation have substantially influenced environmental conditions and ecosystem processes in this region [1,2,3]. As a key component of alpine ecosystems, wetlands provide important ecological functions, including water regulation, biodiversity maintenance, and carbon cycling. However, climate-driven hydrological fluctuations may alter the frequency and duration of waterlogging events, imposing additional environmental constraints on wetland plants [4].

Waterlogging is a major environmental stress affecting plants, particularly in wetland ecosystems where soil oxygen availability is frequently limited. Oxygen deficiency caused by water saturation directly influences root respiration, energy metabolism, and nutrient acquisition, thereby restricting plant growth and performance [5,6]. Roots are particularly vulnerable to waterlogging because oxygen diffusion in flooded soils is substantially slower than in air, resulting in hypoxic or anoxic conditions around the root zone. Consequently, plants have evolved various strategies to regulate root morphological and physiological traits to maintain function under oxygen-limited environments [7,8].

Plant responses to waterlogging are influenced not only by changes in root traits and physiological functions but also by interactions between roots and their surrounding rhizosphere environment. The rhizosphere represents a dynamic interface between plants and soil, where root activity and nutrient transformation processes are closely linked [9]. Waterlogging-induced reductions in soil oxygen availability can alter rhizosphere redox conditions and subsequently influence soil enzyme activities involved in carbon, nitrogen, and phosphorus cycling, including sucrase, urease, and alkaline phosphatase [10]. Changes in root growth under waterlogging may further influence rhizosphere processes under stressful conditions [11,12]. Therefore, evaluating root functional traits together with rhizosphere enzyme activities can help clarify plant belowground responses to waterlogging stress.

Recent transcriptomic studies have shown that plant responses to waterlogging involve changes in oxygen sensing, anaerobic metabolism, reactive oxygen species (ROS) homeostasis, and carbohydrate metabolism. These processes are important for maintaining cellular functions under oxygen-limited conditions [13,14]. In particular, the *ERF-VII*-mediated oxygen sensing pathway and anaerobic metabolism-related genes, including alcohol dehydrogenase (ADH) and pyruvate decarboxylase (*PDC*), are involved in plant responses to hypoxic environments [15,16]. However, most molecular studies have focused on model plants or individual species, and whether plants from habitats with different hydrological conditions exhibit distinct transcriptional responses under waterlogging remains poorly understood. Integrating transcriptomic responses with root functional traits and rhizosphere processes may therefore help explain species-specific responses to waterlogging stress.

*Kobresia tibetica* and *Blysmus sinocompressus* are dominant alpine wetland plants on the Qinghai–Tibet Plateau that occur in habitats with different water availability characteristics. Previous ecological observations suggest that *K. tibetica* is mainly distributed in relatively seasonal wet meadow habitats, whereas *B. sinocompressus* is frequently found in wetter environments. These differences in habitat conditions may contribute to species-specific responses to waterlogging stress. *K. tibetica* is widely distributed in alpine wet meadows where seasonal water fluctuations and temporary waterlogging frequently occur during the growing season, whereas *B. sinocompressus* is frequently found in wetter habitats with greater water availability and stronger hydrological influence. Such differences in long-term hydrological environments may contribute to species-specific responses to waterlogging stress [17].

Therefore, this study selected *K. tibetica* and *B. sinocompressus* as contrasting plant models to investigate species-specific responses of alpine wetland plants to waterlogging stress by integrating root morphology and physiology, rhizosphere enzyme activities, and transcriptomic analyses: (1) Do species from contrasting hydrological habitats exhibit divergent root structural and physiological responses under waterlogging stress? (2) Are changes in rhizosphere functional processes coupled with belowground plant responses during waterlogging? (3) Do species occupying different hydrological habitats employ distinct transcriptional regulatory strategies under waterlogging stress? We hypothesized that the two species would exhibit contrasting waterlogging response strategies, with *B. sinocompressus* maintaining more stable belowground functions and showing stronger regulation of waterlogging-responsive pathways under prolonged waterlogging stress.

## 2. Results

### 2.1. Effects of Waterlogging Stress on Root Morphological Traits

**Figure 1:**
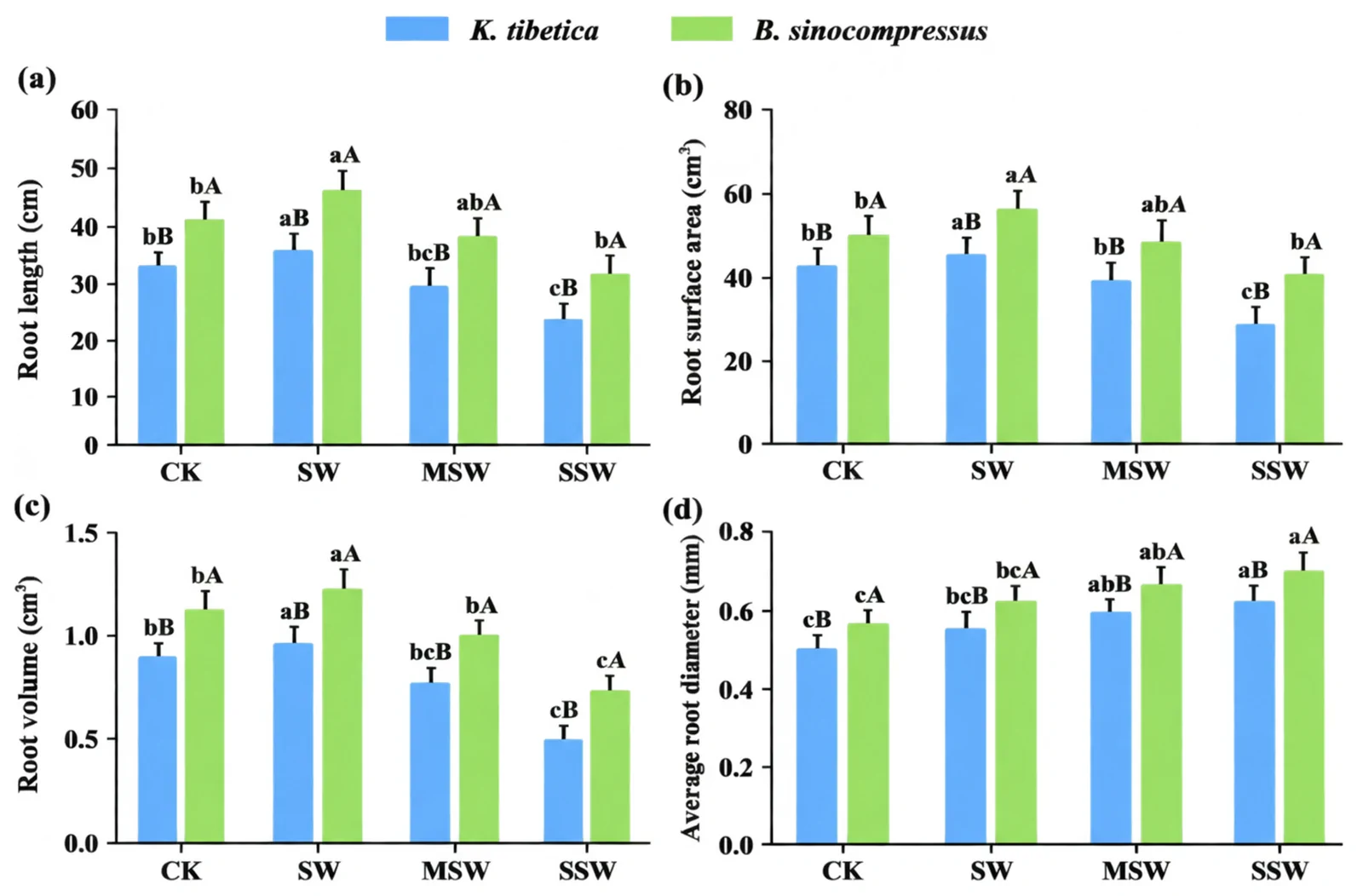
Changes in root morphological traits of *Kobresia tibetica* and *Blysmus sinocompressus* under different waterlogging treatments. CK, control; SW, slight waterlogging; MSW, moderate waterlogging; SSW, severe waterlogging. Different lowercase letters indicate significant differences among waterlogging treatments within each species. Different uppercase letters indicate significant differences between species under the same waterlogging treatment (*p* < 0.05). (**a**) Root length (RL); (**b**) root surface area (RSA); (**c**) root volume (RV); (**d**) average root diameter (ARD).

Waterlogging treatments markedly altered root morphological traits in both plant species (Figure 1). With increasing waterlogging intensity, root length (RL), root surface area (RSA), and root volume (RV) generally showed a slight increase under SW treatment, followed by progressive decreases under MSW and SSW treatments. Under severe waterlogging (SSW), RL, RSA, and RV decreased by 30.5%, 31.8%, and 39.3%, respectively, in *Kobresia tibetica* compared with CK, whereas the corresponding reductions in *Blysmus sinocompressus* were 23.9%, 23.6%, and 29.5%, respectively. The reductions in RL, RSA, and RV under MSW and SSW treatments were greater in *K. tibetica* than in *B. sinocompressus*, indicating stronger inhibition of root growth in *K. tibetica* under moderate and severe waterlogging. In contrast, average root diameter (ARD) exhibited an increasing trend with increasing waterlogging intensity in both species and reached the highest values under SSW treatment. The increased ARD under severe waterlogging indicated that both species exhibited thicker roots under severe waterlogging conditions. Overall, *B. sinocompressus* showed smaller reductions in root morphological traits under moderate and severe waterlogging, whereas *K. tibetica* exhibited stronger decreases in belowground growth-related traits.

### 2.2. Effects of Waterlogging Stress on Root Physiological Traits of Two Plant Species

**Figure 2:**
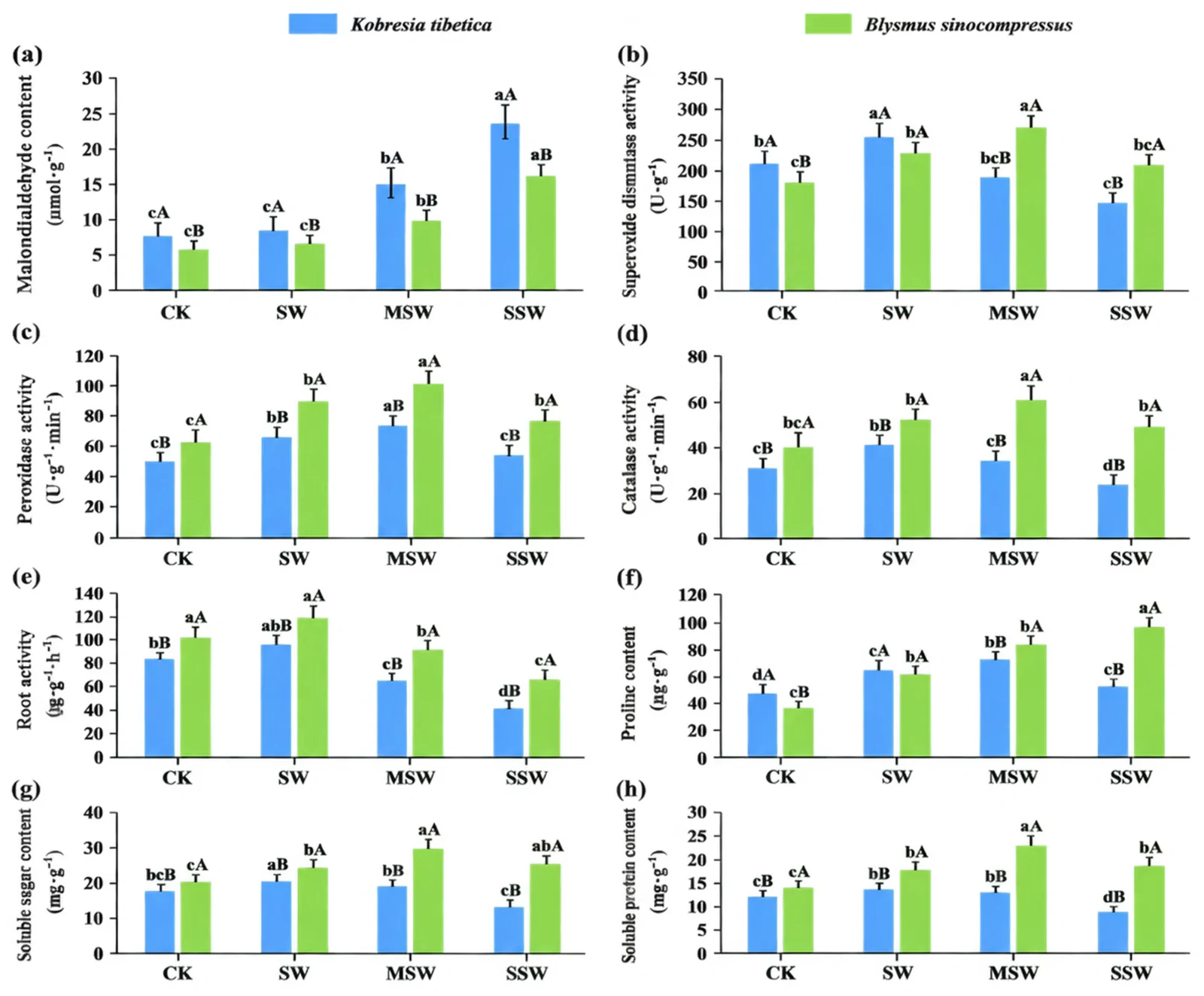
Changes in root physiological traits of *Kobresia tibetica* and *Blysmus sinocompressus* under different waterlogging treatments. CK, control; SW, slight waterlogging; MSW, moderate waterlogging; SSW, severe waterlogging. RA, root activity; MDA, malondialdehyde; SOD, superoxide dismutase; POD, peroxidase; CAT, catalase; Pro, proline; SS, soluble sugar; SP, soluble protein. Different lowercase letters indicate significant differences among treatments within the same species, whereas different uppercase letters indicate significant differences between species under the same treatment (*p* < 0.05). (**a**) Malondialdehyde (MDA) content; (**b**) superoxide dismutase (SOD) activity; (**c**) peroxidase (POD) activity; (**d**) catalase (CAT) activity; (**e**) root activity (RA); (**f**) proline (Pro) content; (**g**) soluble sugar (SS) content; (**h**) soluble protein (SP) content.

Waterlogging treatments markedly affected root physiological traits in both plant species (Figure 2). Root activity (RA) in both species increased under SW treatment but declined with increasing waterlogging intensity under MSW and SSW treatments. Under SW treatment, RA of *Kobresia tibetica* and *Blysmus sinocompressus* increased by 7.7% and 12.9%, respectively, compared with CK, but subsequently decreased under moderate and severe waterlogging. Under SSW treatment, RA decreased by 55.4% and 41.7% in *K. tibetica* and *B. sinocompressus*, respectively, compared with CK. In contrast, malondialdehyde (MDA) content increased progressively with increasing waterlogging intensity. Under SSW treatment, MDA contents in *K. tibetica* and *B. sinocompressus* increased by 229.2% and 150.8%, respectively, compared with CK, and MDA accumulation was lower in *B. sinocompressus* than in *K. tibetica*. Antioxidant enzyme activities showed species-specific responses under different waterlogging conditions. In *K. tibetica*, SOD activity increased under SW treatment but declined under SSW treatment, whereas *B. sinocompressus* maintained higher SOD activity under moderate and severe waterlogging conditions. POD and *CAT* activities exhibited similar trends, with enhanced activities under MSW followed by reductions under SSW, although the magnitude of responses differed between the two species. Osmotic adjustment-related compounds also displayed contrasting responses between species. In *K. tibetica*, Pro, SS, and SP contents increased under SW and MSW treatments but declined under SSW treatment. In contrast, these compounds in *B. sinocompressus* generally increased with increasing waterlogging intensity and reached their highest levels under SSW treatment. Collectively, *B. sinocompressus* maintained higher levels of root activity, antioxidant enzyme activities, and osmotic adjustment substances under prolonged waterlogging, whereas *K. tibetica* showed greater changes in these physiological parameters under severe waterlogging.

### 2.3. Effects of Waterlogging Stress on Rhizosphere Soil Enzyme Activities

**Figure 3:**
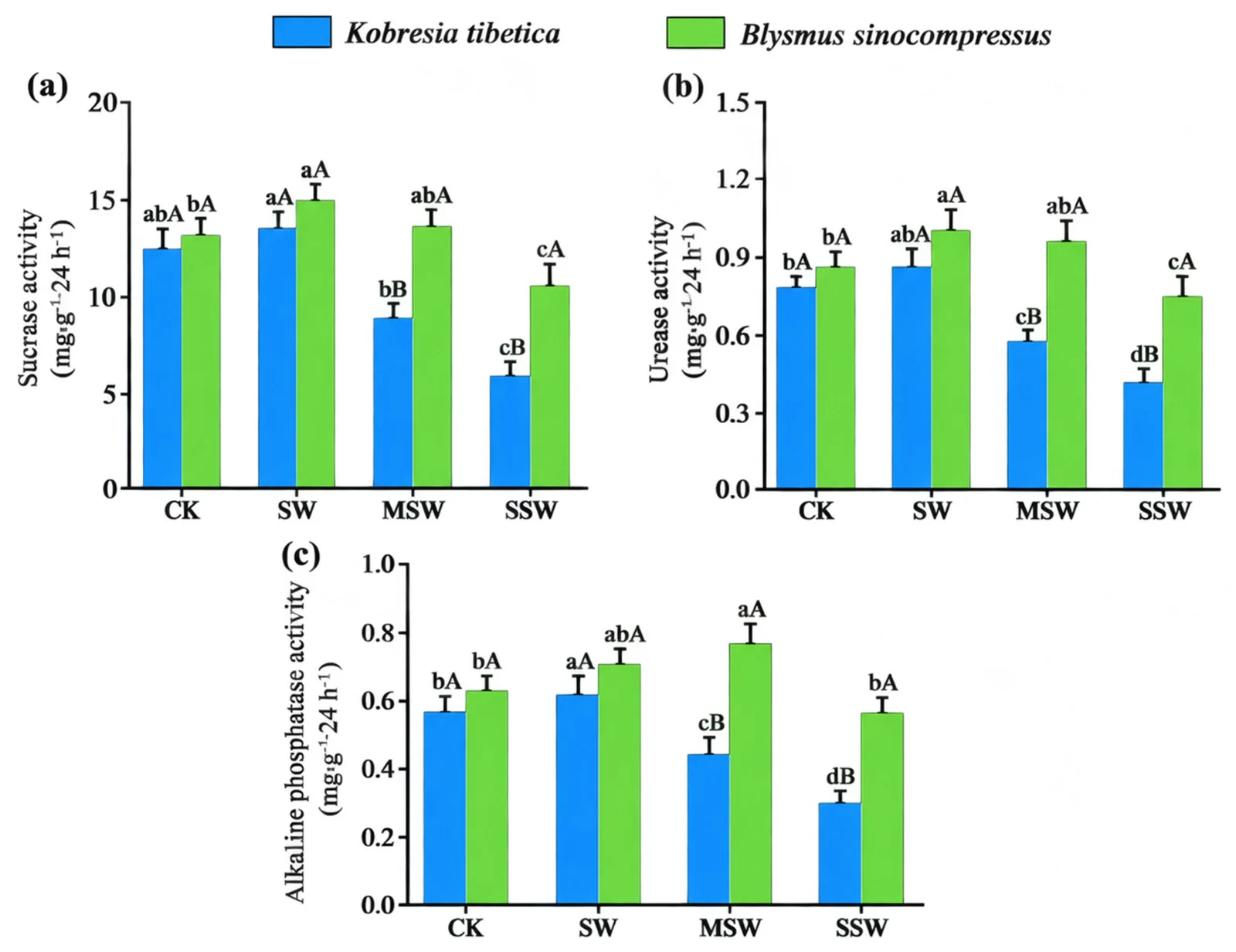
Changes in rhizosphere soil enzyme activities of *Kobresia tibetica* and *Blysmus sinocompressus* under different waterlogging treatments. (**a**) Sucrase (SC) activity; (**b**) urease (URE) activity; (**c**) alkaline phosphatase (ALP) activity. CK, control; SW, slight waterlogging; MSW, moderate waterlogging; SSW, severe waterlogging. Different lowercase letters indicate significant differences among waterlogging treatments within each species; different uppercase letters indicate significant differences between species under the same treatment (*p* < 0.05).

Waterlogging treatments affected rhizosphere soil enzyme activities in both plant species (Figure 3). Rhizosphere sucrase (SC), urease (URE), and alkaline phosphatase (ALP) activities showed species-specific responses under different waterlogging treatments. In *Kobresia tibetica*, SC activity increased by 7.3% under SW treatment compared with CK but decreased progressively under MSW and SSW treatments, with reductions of 23.6% and 49.6%, respectively. Similarly, URE activity increased slightly under SW treatment but declined under MSW and SSW treatments, decreasing by 24.4% and 47.4%, respectively, compared with CK. ALP activity showed a comparable pattern, increasing by 8.9% under SW treatment but decreasing by 23.2% and 50.0% under MSW and SSW treatments, respectively. In *Blysmus sinocompressus*, SC activity increased by 16.4% under SW treatment compared with CK and subsequently declined with increasing waterlogging intensity, with a reduction of 21.6% under SSW treatment. URE activity reached the highest value under SW treatment (1.01 mg g^−1^ 24 h^−1^) and declined to 0.67 mg g^−1^24 h^−1^ under SSW treatment. In contrast to *K. tibetica*, ALP activity in *B. sinocompressus* increased under SW and MSW treatments, reaching the highest value under MSW treatment (0.76 mg g^−1^24 h^−1^), before declining under SSW treatment. Overall, slight waterlogging increased rhizosphere enzyme activities in both species, whereas prolonged waterlogging inhibited enzyme activities. Compared with *K. tibetica*, *B. sinocompressus* exhibited smaller reductions in SC, URE, and ALP activities under moderate and severe waterlogging treatments, indicating greater stability of rhizosphere enzyme activities in *B. sinocompressus* under moderate and severe waterlogging.

### 2.4. Correlation Analysis Among Root Traits, Physiological Responses, and Rhizosphere Soil Enzyme Activities

**Figure 4:**
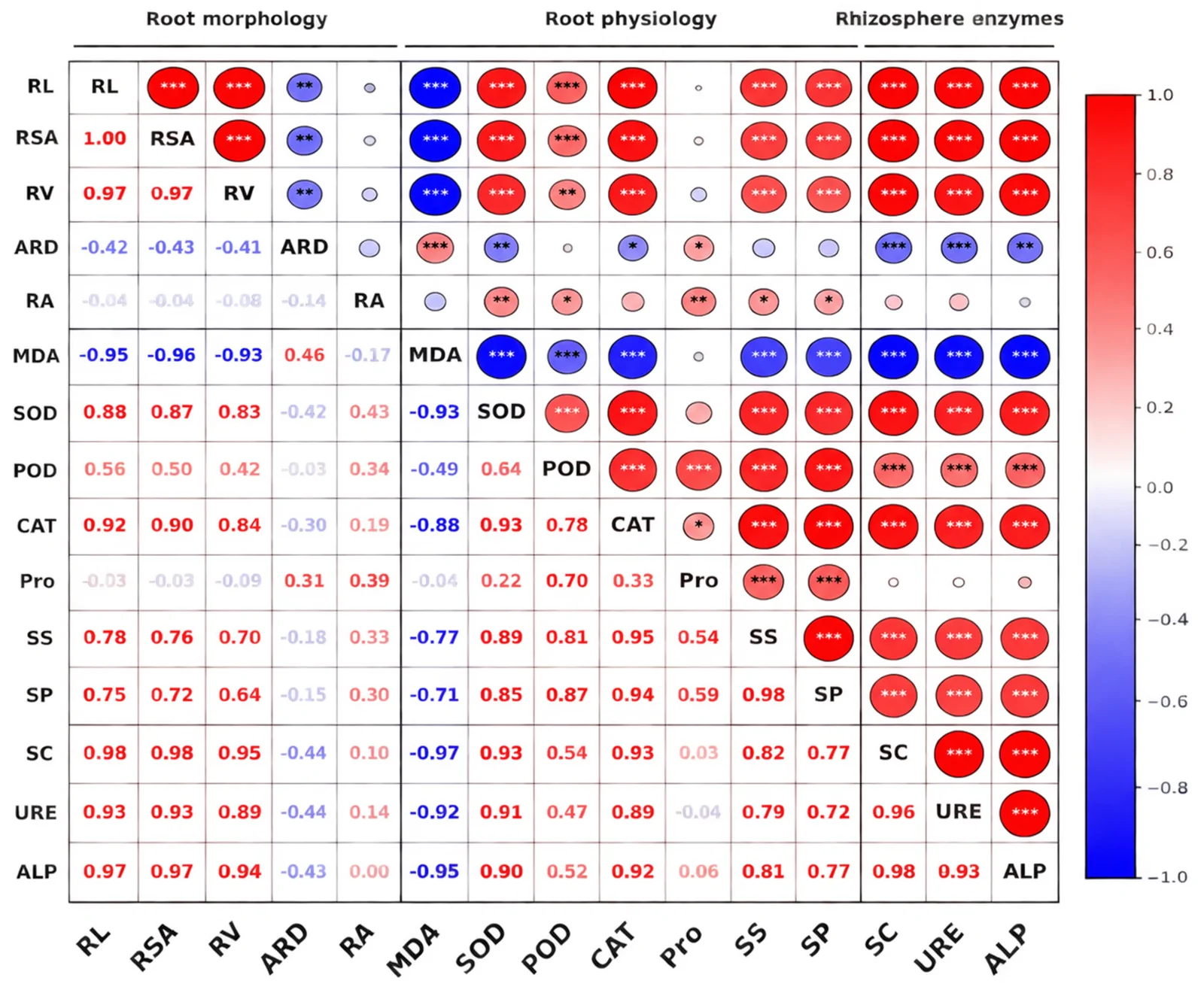
Pearson correlation analysis among root traits, physiological responses, and rhizosphere soil enzyme activities of *Kobresia tibetica* and *Blysmus sinocompressus* under waterlogging stress. Pearson correlation analysis was performed using pooled datasets from the two species. RL, root length; RSA, root surface area; RV, root volume; RA, root activity; MDA, malondialdehyde; SOD, superoxide dismutase; POD, peroxidase; CAT, catalase; Pro, proline; SS, soluble sugar; SP, soluble protein; SC, sucrase; URE, urease; ALP, alkaline phosphatase. One, two, and three asterisks indicate significance at *p* < 0.05, *p* < 0.01, and *p* < 0.001, respectively. The circle size represents the absolute value of Pearson correlation coefficients, with larger circles indicating stronger correlations. The color gradient represents positive and negative correlations.

Pearson correlation analysis was performed using pooled datasets from *Kobresia tibetica* and *Blysmus sinocompressus* to evaluate overall relationships among root traits, physiological responses, and rhizosphere soil enzyme activities under waterlogging conditions (Figure 4). Strong positive correlations were observed among root morphological traits, with RL, RSA, and RV showing significant positive correlations. Root morphological traits were also positively associated with root activity (RA) and several rhizosphere soil enzyme activities. Specifically, RL, RSA, and RV were positively correlated with sucrase (SC), urease (URE), and alkaline phosphatase (ALP) activities. In contrast, malondialdehyde (MDA) exhibited significant negative correlations with most root growth-related traits and rhizosphere soil enzyme activities. Antioxidant enzymes (SOD, POD, and *CAT*) were positively associated with several osmotic adjustment substances and rhizosphere soil enzyme activities. These results revealed overall associations among root traits, physiological responses, and rhizosphere functional processes under waterlogging stress, although the correlations should be interpreted with consideration of species-specific responses.

### 2.5. Principal Component Analysis Reveals Integrated Root and Rhizosphere Responses of Two Alpine Wetland Plants Under Waterlogging Stress

**Figure 5:**
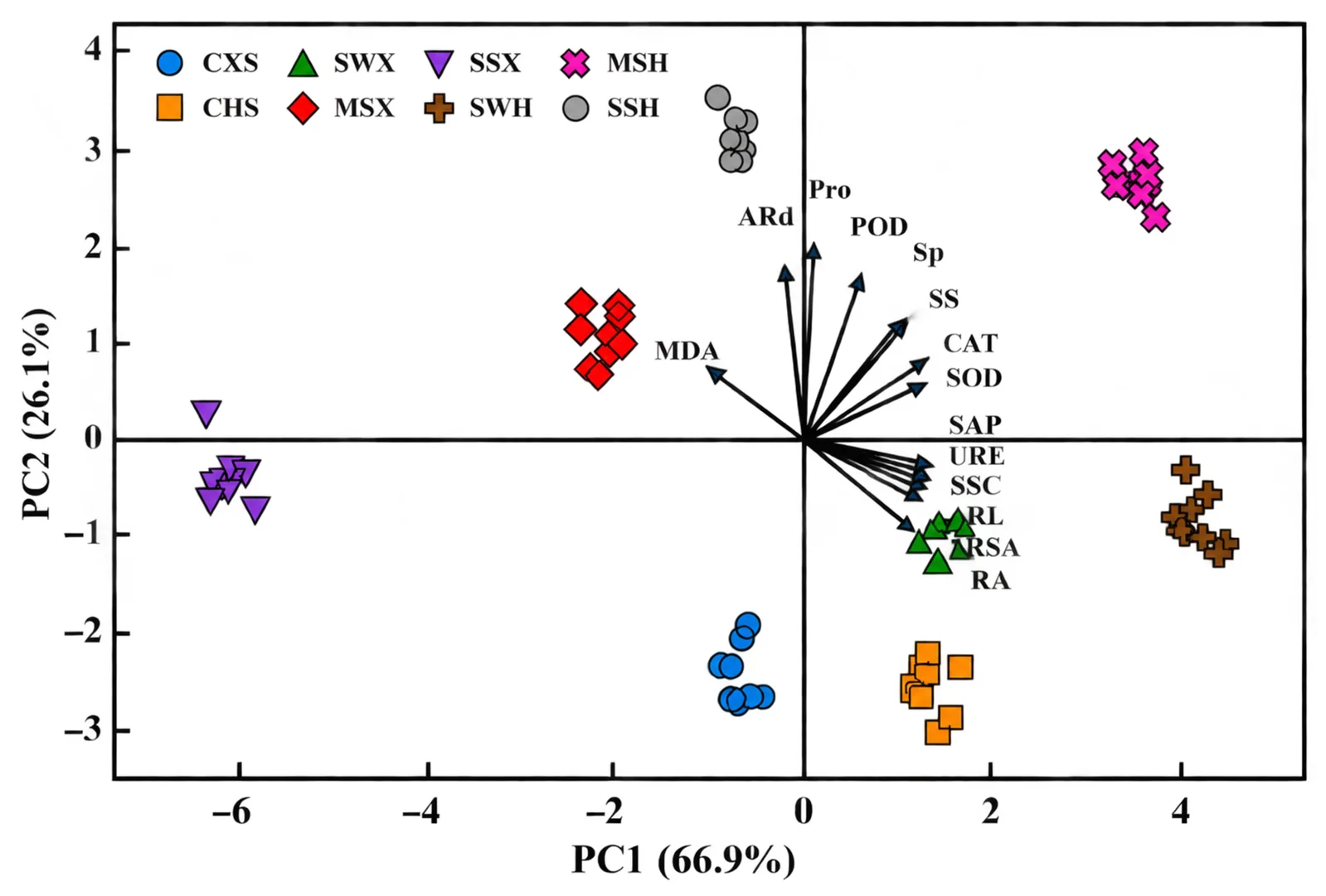
Principal component analysis of root traits, physiological responses, and rhizosphere soil enzyme activities of *Kobresia tibetica* and *Blysmus sinocompressus* under waterlogging stress. PC1 and PC2 explained 66.9% and 26.1% of the total variance, respectively, with a cumulative explained variance of 92.9%. RL, root length; RSA, root surface area; RV, root volume; ARD, average root diameter; RA, root activity; MDA, malondialdehyde; SOD, superoxide dismutase; POD, peroxidase; CAT, catalase; Pro, proline; SS, soluble sugar; SP, soluble protein; SC, sucrase; URE, urease; ALP, alkaline phosphatase. CXS, SWX, MSX, and SSX represent CK, SW, MSW, and SSW treatments of *K. tibetica*, respectively; CHS, SWH, MSH, and SSH represent the corresponding treatments of *B. sinocompressus*.

Principal component analysis (PCA) showed that PC1 and PC2 explained 66.9% and 26.1% of the total variance, respectively, together accounting for 92.9% of the cumulative variation (Figure 5). These two principal components captured the overall variation patterns of root traits, physiological responses, and rhizosphere enzyme activities under different waterlogging treatments. Samples from different plant species and waterlogging treatments exhibited clear separation along the PC1-PC2 axes. In *Kobresia tibetica*, samples from different treatments were gradually distributed along the PC1 axis, with SSW samples clearly separated from CK and SW samples, indicating distinct multivariate response patterns under increasing waterlogging intensity. Samples of *Blysmus sinocompressus* under different waterlogging treatments also showed spatial separation, although the distribution pattern differed from that of *K. tibetica*. Loading analysis showed that root morphological traits (RL, RSA, and RV), root activity (RA), and rhizosphere enzyme activities (SC, URE, and ALP) showed strong positive loadings on PC1, whereas MDA showed a negative loading. Antioxidant enzymes (SOD, POD, and CAT) and osmotic adjustment substances (Pro, SS, and SP) also contributed substantially to the PCA structure. Overall, PCA revealed distinct integrated response patterns between *K. tibetica* and *B. sinocompressus* under waterlogging stress.

### 2.6. Transcriptomic Responses of Roots Under Waterlogging Stress

#### 2.6.1. Quality Assessment of Transcriptome Sequencing Data

RNA sequencing generated high-quality transcriptomic datasets from root samples of *Kobresia tibetica* and *Blysmus sinocompressus* under CK and MSW treatments (Table 1). Considering the cost and complexity of transcriptome sequencing, moderate waterlogging (MSW) was selected as the representative stress condition for RNA-seq analysis. MSW represents an intermediate stage between slight waterlogging-induced acclimation and severe waterlogging-induced functional inhibition, which allows identification of key regulatory pathways associated with waterlogging adaptation. After quality filtering, high-quality clean reads were obtained for all samples. The high proportions of Q20 and Q30 bases, together with the low sequencing error rates, indicated reliable sequencing quality and suitability for subsequent transcriptome analyses. The consistent sequencing quality among biological replicates further supported the reliability of the transcriptomic datasets.

#### 2.6.2. Identification and Distribution of Differentially Expressed Genes

**Figure 6:**
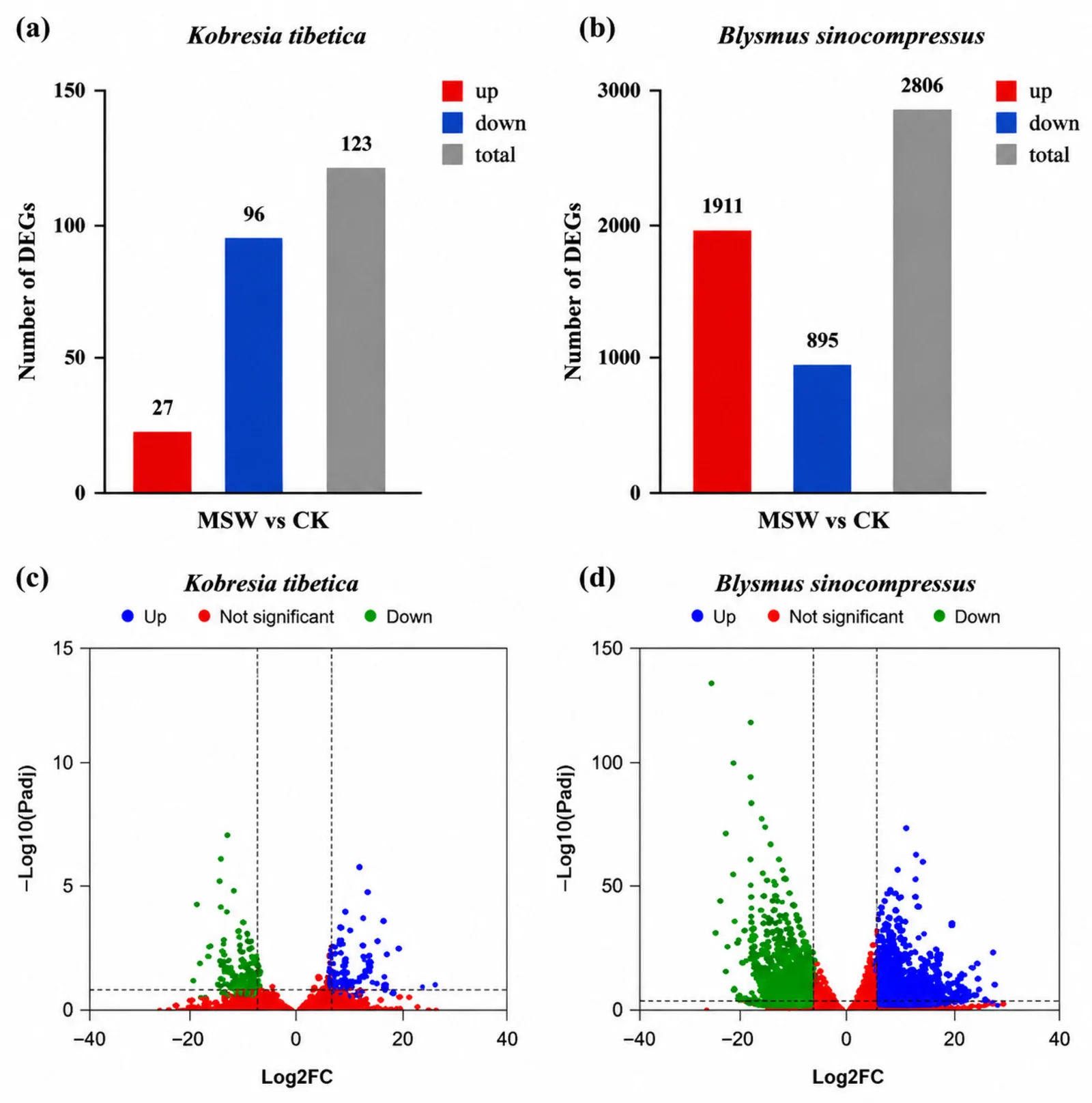
Differentially expressed genes identified in roots of *Kobresia tibetica* and *Blysmus sinocompressus* under moderate waterlogging stress. The numbers of differentially expressed genes (*DEGs*) under moderate waterlogging stress (MSW) compared with the control (CK) are shown in bar charts (**a**,**b**). Red, blue, and gray bars represent upregulated DEGs, downregulated DEGs, and total DEGs, respectively. Volcano plots (**c**,**d**) show the distribution and significance of DEGs between MSW and CK treatments. The *x*-axis represents log_2_(Fold change), and the *y*-axis represents −log_10_(adjusted *p*value). Blue, green, and red dots indicate upregulated, downregulated, and nonsignificant genes, respectively. The dotted horizontal and vertical lines in the volcano plots indicate the adjusted *p*-value and log_2_(fold change) thresholds used to classify DEGs (adjusted *p* < 0.05 and |log_2_(fold change)| ≥ 1).

Differential expression analysis showed different patterns of gene expression changes between *Kobresia tibetica* and *Blysmus sinocompressus* under moderate waterlogging stress (MSW) compared with the control (CK) (Figure 6). In *K. tibetica*, 123 differentially expressed genes (DEGs) were identified, including 27 upregulated and 96 downregulated genes. In contrast, *B. sinocompressus* showed a much larger number of DEGs, with 2806 genes detected, including 1911 upregulated and 895 downregulated genes. The number of DEGs in *B. sinocompressus* was markedly higher than that in *K. tibetica*, suggesting stronger transcriptional changes in response to moderate waterlogging stress.

The volcano plots further showed different DEG distribution patterns between the two species (Figure 6). In *K. tibetica*, most DEGs were distributed close to the center of the plot, and downregulated genes accounted for a larger proportion of the identified DEGs. In *B. sinocompressus*, DEGs were distributed over a wider range of log_2_FC values, with both upregulated and downregulated genes showing greater changes in expression. These results indicate that the two species differed substantially in their transcriptional responses to moderate waterlogging stress.

### 2.7. Functional Enrichment Analysis of Differentially Expressed Genes

**Figure 7:**
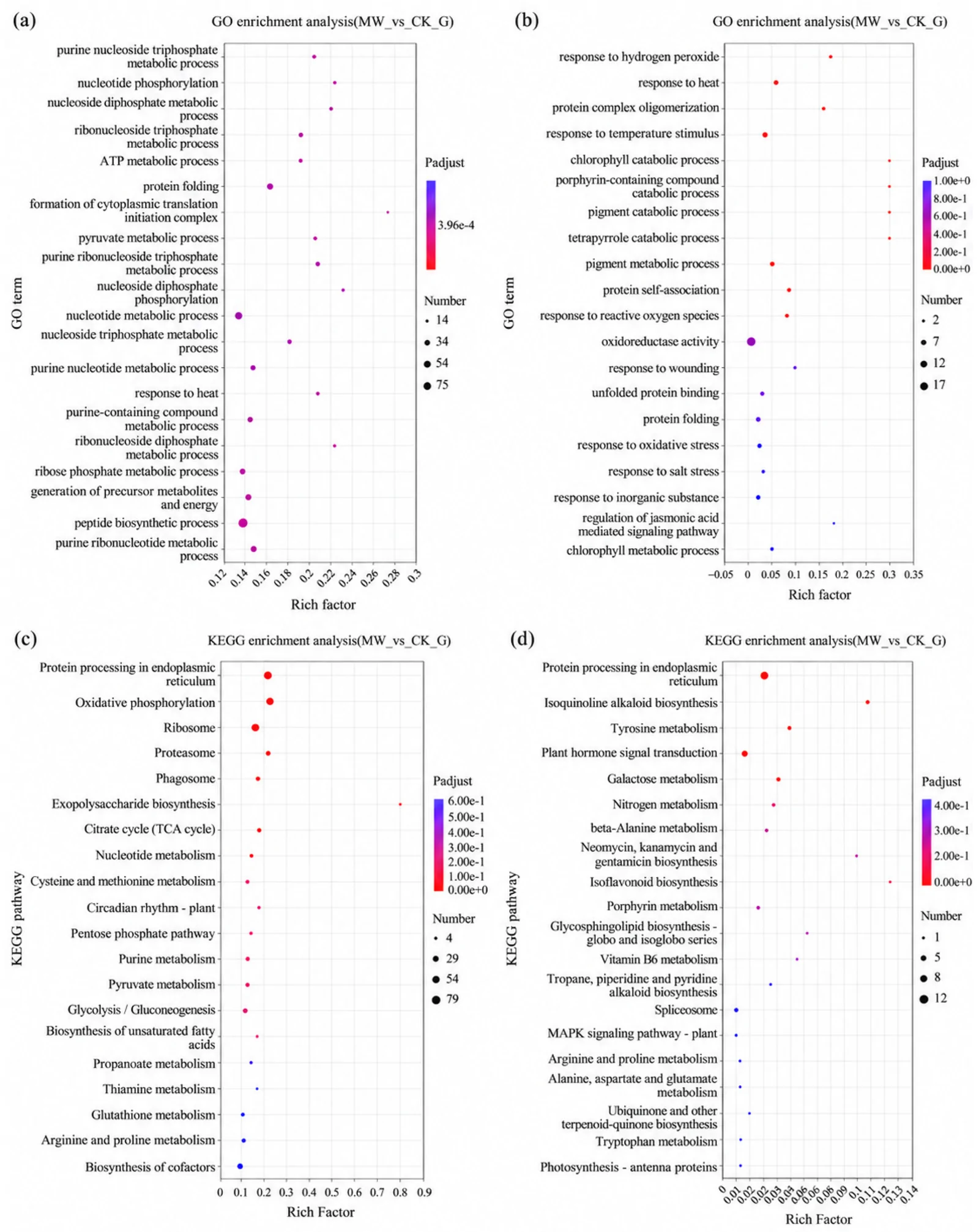
GO and KEGG enrichment analyses of differentially expressed genes in roots of *Kobresia tibetica* and *Blysmus sinocompressus* under moderate waterlogging stress. Panels (**a**,**b**) show GO enrichment results for *B. sinocompressus* and *K. tibetica*, respectively; panels (**c**,**d**) show the corresponding KEGG enrichment results. Bubble size indicates the number of DEGs, whereas bubble color represents the adjusted *p*-value.

GO and KEGG enrichment analyses were performed to characterize the functions and pathways enriched among DEGs under moderate waterlogging stress (Figure 7). GO enrichment analysis showed that DEGs in both species were enriched in multiple functional categories, including biological process (BP), molecular function (MF), and cellular component (CC). In *Kobresia tibetica*, DEGs were mainly enriched in categories related to hydrogen peroxide response, reactive oxygen species (ROS) response, oxidation–reduction processes, protein folding, and stress responses, suggesting that these enriched categories were mainly associated with redox regulation and stress-related processes. In contrast, DEGs in *Blysmus sinocompressus* were enriched in a wider range of functional categories, including cellular metabolic processes, energy-related processes, protein synthesis-related processes, and stress responses.

KEGG pathway enrichment analysis further revealed differences in enriched pathways between the two species. In *K. tibetica*, DEGs were mainly associated with protein processing in the endoplasmic reticulum, plant hormone signal transduction, nitrogen metabolism, arginine and proline metabolism, MAPK signaling pathway, and photosynthesis-antenna proteins. These pathways were mainly related to stress signaling, protein processing, and metabolic regulation. In *B. sinocompressus*, DEGs were enriched in pathways associated with carbon metabolism, glycolysis/gluconeogenesis, pyruvate metabolism, nucleotide metabolism, and glutathione metabolism. These results indicated enrichment of a broader range of metabolic pathways in *B. sinocompressus* under moderate waterlogging stress. Overall, GO and KEGG analyses revealed different functional enrichment patterns between the two species, with *K. tibetica* showing predominant enrichment of stress-related processes, whereas *B. sinocompressus* showed more extensive enrichment of metabolic pathways. Based on these enriched functional pathways, several representative genes associated with hypoxia response, antioxidant regulation, and carbohydrate metabolism were selected for further qRT-PCR validation.

GO enrichment analysis (Figure 7a,b) shows the functional categories enriched among differentially expressed genes (*DEGs*), including biological process (BP), cellular component (CC), and molecular function (MF). KEGG pathway enrichment analysis (Figure 7c,d) illustrates significantly enriched metabolic and signaling pathways associated with DEGs. Bubble size represents the number of DEGs involved in each GO term or KEGG pathway, and bubble color indicates the significance level based on the adjusted *p* value.

### 2.8. Validation of Transcriptome-Derived Waterlogging-Responsive Genes by qRT-PCR

**Figure 8:**
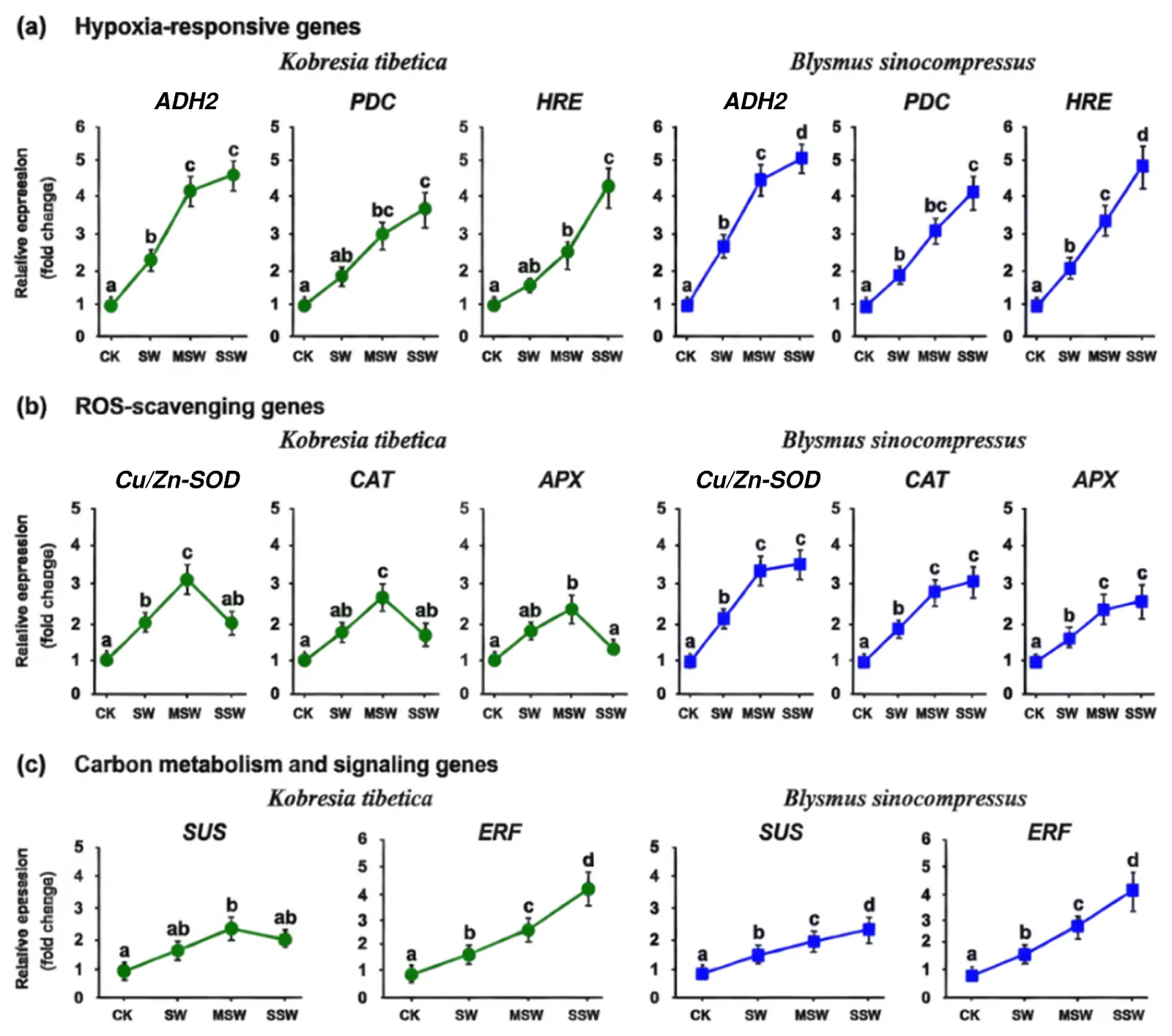
Expression patterns of key waterlogging-responsive genes in roots of *Kobresia tibetica* and *Blysmus sinocompressus* validated by quantitative reverse transcription polymerase chain reaction (qRT-PCR). (**a**) Hypoxia-responsive and anaerobic metabolism-related genes, including alcohol dehydrogenase 2 (*ADH2*), pyruvate decarboxylase (*PDC*), and hypoxia-responsive gene (*HRE*); (**b**) antioxidant defense-related genes, including Cu/Zn-superoxide dismutase (*Cu/Zn-SOD*), catalase (*CAT*), and ascorbate peroxidase (*APX*); (**c**) carbohydrate metabolism-related gene sucrose synthase (*SUS*) and stress signaling-related gene ethylene-responsive factor (*ERF*). Relative gene expression levels were calculated using the 2^−ΔΔCt^ method. Values are presented as means ± SD (n = 3). Different lowercase letters indicate significant differences among waterlogging treatments within the same species (*p* < 0.05).

Based on the differentially expressed genes identified by RNA-seq and their functional enrichment results, representative genes involved in hypoxia response, anaerobic metabolism, antioxidant defense, and carbohydrate metabolism were selected for qRT-PCR validation. These genes were selected because they represent key regulatory components associated with waterlogging responses and showed significant transcriptional changes in RNA-seq analysis. The qRT-PCR results showed similar expression trends to the RNA-seq results (Figure 8), supporting the reliability of transcriptomic analysis. Furthermore, qRT-PCR analysis revealed the dynamic expression patterns of these transcriptome-derived candidate genes under different waterlogging intensities (SW, MSW, and SSW), demonstrating species-specific transcriptional regulation during progressive waterlogging stress. Hypoxia-responsive and anaerobic metabolism-related genes, including hypoxia-responsive gene (*HRE*), alcohol dehydrogenase 2 (*ADH2*), and pyruvate decarboxylase (*PDC*), exhibited dynamic expression patterns under different waterlogging intensities in both species. Compared with *Kobresia tibetica*, *Blysmus sinocompressus* showed stronger induction of these genes under moderate and severe waterlogging conditions, indicating divergent transcriptional responses to waterlogging-induced hypoxia between the two species. The ethylene-responsive factor (*ERF*) gene also showed differential expression patterns under different waterlogging intensities, with stronger induction observed in *B. sinocompressus* under moderate and severe waterlogging conditions. Antioxidant defense-related genes, including Cu/Zn-superoxide dismutase (*Cu/Zn-SOD*), catalase (*CAT*), and ascorbate peroxidase (*APX*), displayed species-specific expression patterns. In *K. tibetica,* the expression levels of these genes increased under SW and MSW treatments but decreased under SSW treatment. In contrast, *B. sinocompressus* maintained stronger transcriptional activation of antioxidant defense-related genes under severe waterlogging conditions. The carbohydrate metabolism-related gene sucrose synthase (*SUS*) was also induced under different waterlogging intensities. Its expression showed distinct responses between the two species, with relatively stronger induction observed in *B. sinocompressus* under moderate and severe waterlogging conditions. Overall, qRT-PCR validation supported the expression patterns identified by RNA-seq. More importantly, the analysis across different waterlogging intensities revealed that these transcriptome-derived candidate genes exhibited dynamic and species-specific regulation during progressive waterlogging stress.

## 3. Discussion

### 3.1. Differential Root Structural Responses of Plants with Different Hydrological Niches to Waterlogging Stress

Roots are among the first organs affected by oxygen limitation during waterlogging and play important roles in maintaining plant growth and resource acquisition. Previous studies have shown that reduced oxygen availability restricts root respiration and energy metabolism, thereby inhibiting root growth and function under prolonged waterlogging [18,19]. However, plant species from different hydrological environments may differ in their capacity to maintain root performance under waterlogged conditions [19]. In the present study, slight waterlogging promoted increases in root length (RL), root surface area (RSA), and root volume (RV) in both species, whereas these traits decreased under moderate and severe waterlogging. Similar responses have been reported in other plants, where moderate water availability can stimulate root growth, while prolonged hypoxia restricts root development due to oxygen deficiency [20,21]. Compared with *Kobresia tibetica*, *Blysmus sinocompressus* maintained more stable root morphological traits under prolonged waterlogging, with smaller reductions in RL, RSA, and RV. This difference may be related to the contrasting hydrological environments experienced by the two species in their natural habitats. Species occurring in wetter habitats may have developed greater capacity to maintain root functions under moderate and severe soil saturation [22,23], which may contribute to the relatively stable root system of *B. sinocompressus* under moderate and severe waterlogging. Average root diameter (ARD) increased with increasing waterlogging intensity in both species. Similar increases in root thickness have been reported as a morphological adjustment under unfavorable soil conditions [24,25]. The increase in ARD observed in this study may represent a common morphological response that contributes to maintaining root morphological stability under oxygen-limited conditions. Overall, the two species exhibited different root structural response strategies under waterlogging stress, and the greater stability of *B. sinocompressus* suggests that contrasting hydrological niches may contribute to species-specific responses to waterlogging.

### 3.2. Root Physiological Regulation and Redox Homeostasis Responses Under Waterlogging Stress

Waterlogging-induced oxygen limitation affects root respiration and cellular redox balance. Under hypoxic conditions, excessive reactive oxygen species (ROS) accumulation can promote membrane lipid peroxidation and cellular damage when ROS production exceeds antioxidant capacity. Therefore, antioxidant regulation is essential for maintaining cellular homeostasis under waterlogging stress [12,26]. In the present study, malondialdehyde (MDA) content increased progressively with increasing waterlogging intensity in both species, whereas *Kobresia tibetica* showed greater MDA accumulation under severe waterlogging, accompanied by a stronger decline in root activity. This pattern agrees with previous studies showing that hypoxic stress promotes ROS accumulation and enhances membrane lipid peroxidation [27,28]. Similar increases in MDA content under waterlogging have also been reported in other plant species, although the magnitude of oxidative damage varies among species with different stress response capacities. In contrast, *Blysmus sinocompressus* maintained lower MDA levels under the same conditions, suggesting relatively lower oxidative damage and more effective redox regulation. Previous studies have suggested that plants occurring in waterlogged habitats may possess more effective regulatory mechanisms to alleviate oxidative stress under prolonged waterlogging [29,30]. Thus, the lower MDA accumulation in *B. sinocompressus* may contribute to its greater physiological stability under severe waterlogging. Antioxidant enzyme activities further reflected differences in ROS regulation between the two species. In this study, SOD, POD, and CAT activities increased under slight and moderate waterlogging but showed different responses under severe waterlogging. Similar patterns have been reported in waterlogging-stressed plants, where antioxidant enzymes are activated during early stress stages but may decline when prolonged hypoxia exceeds the capacity of protective systems. ROS are not only toxic molecules causing oxidative damage but also function as signaling molecules involved in stress responses, requiring plants to maintain a balance between ROS production and scavenging [31,32,33]. In *K. tibetica*, the decline in antioxidant enzyme activities together with increased MDA accumulation under severe waterlogging suggests that ROS-scavenging capacity may have become insufficient to counteract oxidative stress under prolonged hypoxia. In contrast, *B. sinocompressus* maintained relatively higher antioxidant enzyme activities, indicating different strategies for regulating oxidative stress between the two species. Osmotic adjustment also contributed to species-specific physiological responses under waterlogging. Proline, soluble sugars, and soluble proteins participate in osmotic regulation and metabolic maintenance under stress conditions [34,35]. In the present study, *B. sinocompressus* maintained higher levels of Pro, SS, and SP under waterlogging conditions, whereas these compounds declined in *K. tibetica* under severe waterlogging. Previous studies have shown that accumulation of osmoprotective compounds and regulation of carbohydrate metabolism can help plants maintain metabolic stability under hypoxic conditions [36]. Therefore, the stronger maintenance of osmotic adjustment capacity in *B. sinocompressus* may help sustain cellular protection and physiological activity during prolonged waterlogging. Overall, both species responded to waterlogging through antioxidant regulation and osmotic adjustment, but their regulatory capacities differed. *K. tibetica* experienced greater oxidative stress and impaired physiological regulation under severe waterlogging, whereas *B. sinocompressus* maintained relatively stable redox balance and osmotic regulation. These differences reflect divergent physiological response strategies between the two species with contrasting hydrological niches.

### 3.3. Rhizosphere Functional Responses of Plants with Different Hydrological Niches Under Waterlogging Stress

The rhizosphere represents an important interface where plant roots interact with surrounding soil conditions [37]. Waterlogging not only affects root physiological processes but also modifies rhizosphere biochemical functions by changing soil oxygen availability and redox conditions. Soil enzyme activities are commonly used as indicators of rhizosphere functional changes because they are closely associated with organic matter decomposition and nutrient cycling [38]. Reduced oxygen diffusion under waterlogging can modify soil microenvironmental conditions and subsequently influence enzyme activities involved in carbon, nitrogen, and phosphorus cycling [39,40].

In the present study, waterlogging significantly altered rhizosphere sucrase (SC), urease (URE), and alkaline phosphatase (ALP) activities in both species [41]. In *Kobresia tibetica*, rhizosphere enzyme activities increased under slight waterlogging but gradually declined with increasing waterlogging intensity. In contrast, *Blysmus sinocompressus* maintained relatively higher SC, URE, and ALP activities under moderate and severe waterlogging. Similar patterns have been reported in previous studies, where moderate water availability may temporarily stimulate rhizosphere biochemical processes, whereas prolonged waterlogging and oxygen limitation suppress enzyme activities associated with nutrient cycling [42]. These results suggest that the intensity and duration of waterlogging strongly influence rhizosphere enzyme responses under the conditions tested. The differences in rhizosphere enzyme activities between the two species were closely associated with their root functional responses. In this study, root length (RL), root surface area (RSA), and root volume (RV) showed significant positive correlations with SC, URE, and ALP activities, indicating coordinated changes between root development and rhizosphere nutrient transformation processes. Previous studies have demonstrated that plants may influence rhizosphere biochemical processes through changes in root traits and associated rhizosphere interactions, while rhizosphere enzyme activities reflect adjustments in plant–soil relationships under changing environmental conditions [43]. Under severe waterlogging, *K. tibetica* showed pronounced reductions in rhizosphere enzyme activities, together with decreased root activity and increased oxidative stress. This pattern suggests that prolonged waterlogging may simultaneously constrain root functioning and rhizosphere nutrient transformation processes [44]. In contrast, *B. sinocompressus* maintained relatively stable rhizosphere enzyme activities, consistent with its more stable root morphology and physiological regulation under waterlogging. Overall, the contrasting rhizosphere responses between the two species indicate that differences in hydrological niches are associated with different capacities for maintaining root–rhizosphere functional processes under waterlogging stress.

### 3.4. Divergent Transcriptional Responses of Plants with Different Hydrological Niches Under Waterlogging Stress

Waterlogging reduces oxygen availability in the rhizosphere, requiring plants to regulate gene expression to maintain energy metabolism, redox balance, and cellular homeostasis [45]. Under hypoxic conditions, the *ERF-VII*-mediated hypoxia signaling pathway and anaerobic metabolism-related genes, such as *ADH* and *PDC,* are considered key molecular components of plant responses to waterlogging [46]. However, plant species with different ecological backgrounds may exhibit distinct hypoxia-responsive patterns due to differences in physiological regulation and molecular control mechanisms [47]. In the present study, transcriptome analysis revealed distinct gene expression responses between the two plant species under moderate waterlogging. A total of 123 differentially expressed genes (DEGs) were identified in *Kobresia tibetica*, whereas 2806 DEGs were detected in *Blysmus sinocompressus*, with differences in both the number and direction of transcriptional changes. These results indicate that the two species exhibited contrasting transcriptional response patterns under moderate waterlogging. Previous studies have demonstrated considerable interspecific variation in hypoxia-responsive gene regulation, and the magnitude of transcriptional responses may be associated with species-specific regulatory patterns and ecological characteristics.

Functional enrichment analysis further revealed divergent transcriptional regulation patterns between the two species. In *K. tibetica*, DEGs were mainly associated with oxidation-reduction processes, responses to stimuli, and protein homeostasis-related functions, which were consistent with the oxidative stress responses observed during waterlogging treatments. Because maintenance of redox homeostasis is essential under hypoxic conditions, transcriptional regulation of ROS-related processes represents an important component of plant stress responses [48]. Therefore, the transcriptional changes in *K. tibetica* may reflect a relatively conservative stress-protection response under moderate waterlogging. In contrast, DEGs in *Blysmus sinocompressus* were enriched in broader functional categories, including carbon metabolism, energy metabolism, protein metabolism, and stress responses. Under oxygen deficiency, plants commonly enhance glycolysis and anaerobic fermentation pathways to maintain energy supply, with *ADH* and *PDC* functioning as key enzymes involved in anaerobic metabolism. In the present study, *B. sinocompressus* showed stronger induction of hypoxia-responsive genes validated by qRT-PCR, which was consistent with its higher root activity, lower MDA accumulation, and stronger antioxidant regulation under waterlogging. These coordinated transcriptional responses may contribute to maintaining belowground functional stability under moderate waterlogging. The qRT-PCR results further supported the expression patterns identified by RNA-seq. Hypoxia-responsive genes (*ADH*, *PDC*, *HRE*, and *ERF*), antioxidant-related genes (*SOD*, *CAT*, and *APX*), and the sugar metabolism-related gene (*SUS*) showed species-specific responses to waterlogging. These divergent expression patterns indicate that the two plant species exhibit different molecular response patterns under waterlogging stress, which may be associated with their contrasting hydrological niches and belowground functional characteristics.

### 3.5. Root–Rhizosphere–Transcriptomic Coordination Reveals Differential Waterlogging Response Strategies of Plants with Different Hydrological Niches

Plant responses to waterlogging stress are not determined by a single trait but result from the coordinated regulation of root architecture, physiological processes, rhizosphere functions, and molecular responses [37]. In the present study, integrated analyses of root traits, physiological responses, rhizosphere functions, and transcriptomic profiles revealed that *Kobresia tibetica* and *Blysmus sinocompressus* experienced waterlogging stress but exhibited distinct response patterns. Under prolonged waterlogging, *K. tibetica* showed restricted root growth, increased MDA accumulation, and decreased rhizosphere soil enzyme activities. At the transcriptomic level, its differentially expressed genes were mainly associated with oxidation–reduction regulation and stress-responsive processes. These results suggest that *K. tibetica* mainly exhibited stress-responsive regulation under waterlogging-induced hypoxic conditions, whereas prolonged stress may limit its capacity to maintain belowground functional performance [33]. In contrast, *B. sinocompressus* maintained relatively stable root architecture, physiological status, and rhizosphere enzyme activities under moderate and severe waterlogging conditions. Its differentially expressed genes were associated with carbon metabolism, energy metabolism, and multiple stress-responsive processes. During hypoxia responses, plants generally coordinate root structural adjustment, energy metabolism maintenance, and redox homeostasis regulation to preserve cellular functions [47]. The consistency among root traits, physiological responses, rhizosphere functions, and transcriptomic changes suggests that *B. sinocompressus* may maintain more stable belowground functioning through coordinated regulation across multiple biological levels [48]. Overall, plants with different hydrological niches do not employ identical waterlogging response patterns but instead exhibit distinct regulatory strategies associated with their ecological characteristics. *B. sinocompressus*, which commonly occurs in wetter habitats, showed a greater capacity for maintaining coordination among root, physiological, rhizosphere, and transcriptional responses, whereas *K. tibetica* exhibited stronger stress-responsive regulation under prolonged waterlogging. This study integrates multiple functional levels to reveal divergent belowground response strategies of alpine wetland plants under changing hydrological conditions, providing insights into potential responses of alpine wetland plants to future hydrological fluctuations. However, because only one species was selected to represent each habitat type, the observed differences should be interpreted as species-specific waterlogging responses rather than universal adaptations associated with hydrological conditions. Further comparative studies involving multiple wetland species are needed to determine whether similar response patterns occur across broader ecological gradients.

## 4. Materials and Methods

### 4.1. Plant Materials and Experimental Design

**Figure 9:**
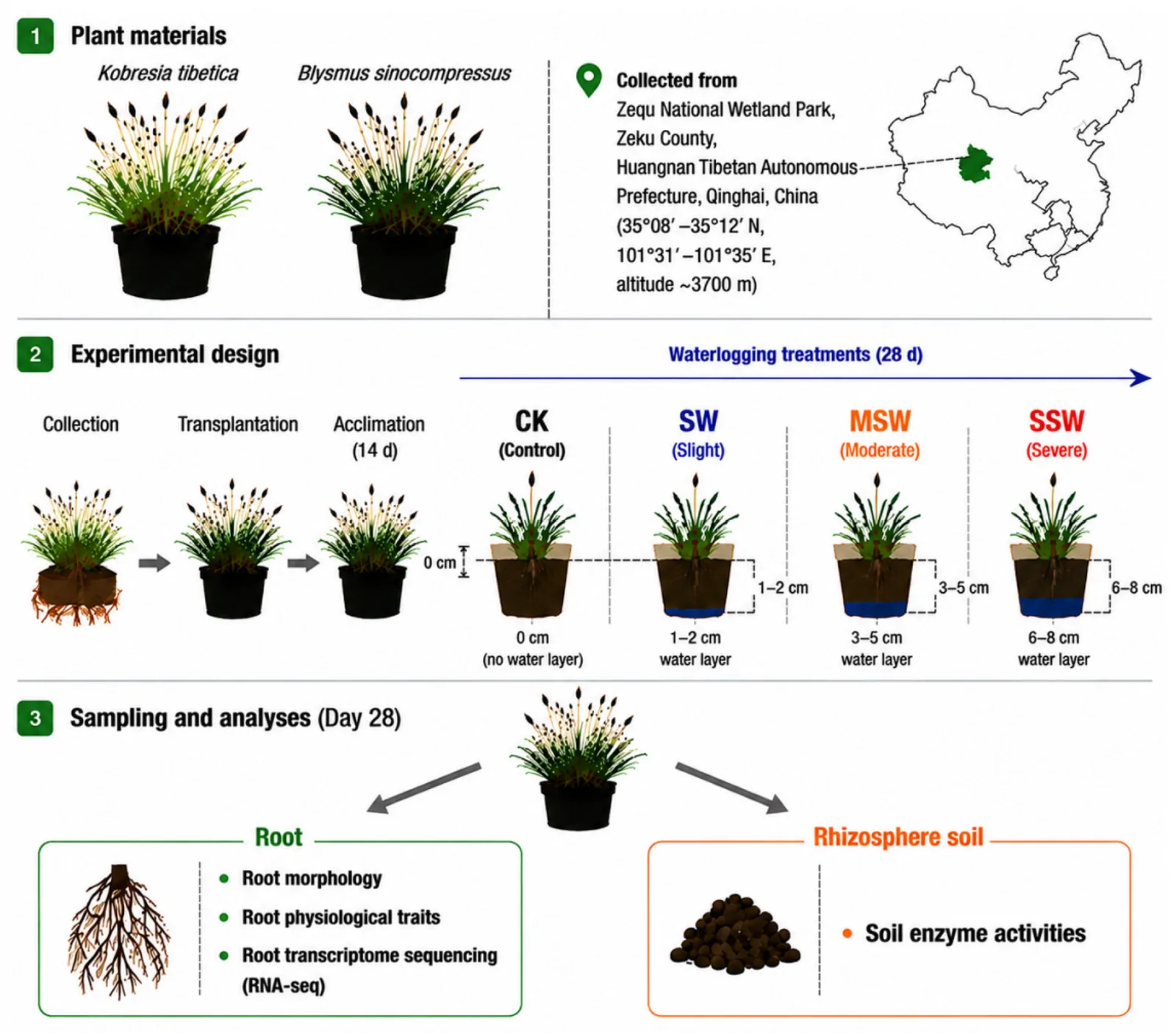
Experimental design and workflow for investigating waterlogging responses in *Kobresia tibetica* and *Blysmus sinocompressus*. Plant materials were collected from Zequ National Wetland Park, Qinghai Province, China. After transplantation and a 14-day acclimation period, plants were subjected to four waterlogging treatments for 28 days: control (CK, 0 cm), slight waterlogging (SW, 1–2 cm), moderate waterlogging (MSW, 3–5 cm), and severe waterlogging (SSW, 6–8 cm). Root and rhizosphere soil samples were collected after the waterlogging treatments for analyses of root morphological traits, physiological responses, root transcriptome sequencing (RNA-seq), and rhizosphere soil enzyme activities. Arrows indicate the progression from collection and acclimation through the 28-day treatments and the subsequent separation of root and rhizosphere soil samples for analysis. Blue indicates the waterlogging-treatment period, green indicates plant/root-related components, and orange indicates rhizosphere-soil-related components.

In June 2025, similarly sized individuals of *Kobresia tibetica* and *Blysmus sinocompressus*, two dominant alpine wetland plants representing contrasting hydrological niches, were collected from Zequ National Wetland Park, Huangnan Tibetan Autonomous Prefecture, Qinghai Province, China (35°08′–35°12′ N, 101°31′–101°35′ E; approximately 3700 m above sea level). Whole plants were carefully excavated with surrounding soil attached to the root systems to minimize root damage during transplantation. The collected plants were transported to the greenhouse of Qinghai University and transplanted into plastic pots (25 cm in diameter × 30 cm in height), with three plants per pot. Soil collected from the 0–20 cm soil layer at the sampling site was used as the potting substrate. After removing visible plant residues and stones, the soil was passed through a 2 mm sieve, thoroughly homogenized, and 10 kg of soil was added to each pot. After transplantation, plants were acclimated for 14 days under greenhouse conditions. During acclimation, temperature was maintained at 25 ± 2 °C, relative humidity at 60–70%, and soil moisture at 70–80% of field capacity. After acclimation, plants were subjected to four waterlogging treatments: control (CK), slight waterlogging (SW), moderate waterlogging (MSW), and severe waterlogging (SSW) (Figure 9). Waterlogging intensity was defined according to the depth of standing water maintained above the soil surface. The CK treatment was maintained without standing water, whereas SW, MSW, and SSW treatments were maintained at water depths of 1–2 cm, 3–5 cm, and 6–8 cm above the soil surface, respectively. These water levels were selected to represent different degrees of seasonal inundation commonly occurring in alpine wetland habitats. Water levels were monitored daily, and evaporative losses were replenished to maintain stable treatment conditions. Waterlogging treatments were maintained for 28 days. A two-factor factorial experiment was conducted using a completely randomized design, with species and waterlogging treatment as the two experimental factors. Six independent biological replicates were established for each species × treatment combination (n = 6). Pot positions were randomly rearranged every three days to minimize potential positional effects within the greenhouse.

### 4.2. Sample Collection and Processing

After 28 days of waterlogging treatments, root and rhizosphere soil samples were collected for subsequent analyses of root morphology, physiological responses, rhizosphere enzyme activities, transcriptome sequencing, and qRT-PCR validation. Six independent biological replicates were randomly selected from each species × treatment combination (n = 6). Plants were carefully removed from the pots and loosely attached soil particles were gently removed from the root surface. Soil tightly adhering to the root surface was collected as rhizosphere soil. After removing visible plant residues and stones, rhizosphere soil samples were passed through a 2 mm sieve and stored at 4 °C for subsequent determination of soil enzyme activities. Root samples were gently washed with deionized water to remove residual soil particles and blotted dry with absorbent paper. Fresh roots were divided into two portions. One portion was immediately used for root morphological measurements and root activity determination, while the other portion was immediately frozen in liquid nitrogen and stored at −80 °C for subsequent physiological and molecular analyses, including determination of osmotic adjustment substances (Pro and SS), soluble protein (SP), oxidative stress indicators (MDA), antioxidant enzyme activities, transcriptome sequencing, and qRT-PCR analysis. Based on preliminary physiological responses and the objective of identifying transcriptional responses associated with waterlogging stress, root samples from CK and MSW treatments were selected for transcriptome sequencing. Three biological replicates were used for each species × treatment combination, resulting in a total of 12 RNA-seq libraries. The MSW treatment was selected because it represented an intermediate waterlogging condition that induced measurable physiological responses while avoiding severe physiological impairment associated with severe waterlogging. Unlike transcriptome sequencing, which focused on a representative intermediate waterlogging condition, qRT-PCR analysis was conducted across all waterlogging gradients to validate the dynamic expression patterns of candidate genes under different waterlogging intensities.

### 4.3. Measurement of Experimental Traits

#### 4.3.1. Measurement of Root Morphological Traits

Fresh roots were carefully washed with deionized water to remove adhering soil particles and gently blotted dry with absorbent paper. The cleaned roots were evenly spread in a transparent scanning tray to minimize overlapping among root segments. Root images were acquired using a root scanner (Epson Expression 12000XL, Seiko Epson Corporation, Suwa, Japan), and root morphological traits were analyzed using WinRHIZO Pro 2009 software (Regent Instruments Inc., Québec, QC, Canada). The measured traits included root length (RL), root surface area (RSA), root volume (RV), and average root diameter (ARD). Each sample was scanned three times, and the mean value was used for subsequent statistical analyses.

#### 4.3.2. Measurement of Root Physiological Traits

Root physiological traits were measured using fresh roots or root samples stored at −80 °C after rapid freezing in liquid nitrogen. Root activity was determined immediately using fresh roots, while the contents of proline (Pro), soluble sugar (SS), soluble protein (SP), and malondialdehyde (MDA), as well as antioxidant enzyme activities, were determined using frozen root samples. All measurements were conducted according to previously described methods [22]. Briefly, root activity was determined using the triphenyltetrazolium chloride (TTC) reduction method. Pro content was determined using the sulfosalicylic acid method, SS content using the anthrone colorimetric method, SP content using the Coomassie brilliant blue method, and MDA content using the thiobarbituric acid (TBA) method. The activities of antioxidant enzymes, including superoxide dismutase (SOD), peroxidase (POD), and catalase (CAT), were determined using the nitroblue tetrazolium (NBT) photoreduction method, guaiacol oxidation method, and hydrogen peroxide decomposition assay, respectively. Six biological replicates were analyzed for each species × treatment combination.

#### 4.3.3. Measurement of Rhizosphere Soil Enzyme Activities

Fresh rhizosphere soil samples were passed through a 2 mm sieve and used for enzyme activity assays. Sucrase (SC) activity was measured using the 3,5-dinitrosalicylic acid (DNS) colorimetric method. Urease (URE) activity was determined using the indophenol blue colorimetric method. Alkaline phosphatase (ALP) activity was measured using the disodium phenyl phosphate colorimetric method. All enzyme activities were determined according to Guan [49] with slight modifications. Enzyme activities were expressed on a dry soil weight basis.

### 4.4. Transcriptome Sequencing and qRT-PCR Analysis

#### 4.4.1. RNA Extraction and Transcriptome Sequencing

Root samples from the CK and MSW treatments were selected for transcriptome sequencing based on the objective of identifying molecular responses under moderate waterlogging stress. Three biological replicates were included for each species × treatment combination, resulting in a total of 12 RNA-seq libraries. Total RNA was extracted from frozen root samples, and RNA concentration and purity were assessed using a NanoDrop spectrophotometer (Thermo Fisher Scientific, Waltham, MA, USA). RNA integrity was evaluated by agarose gel electrophoresis and an Agilent 2100 Bioanalyzer (Agilent Technologies, Santa Clara, CA, USA). Qualified RNA samples were used for cDNA library construction and sequencing on an Illumina high-throughput sequencing platform (Illumina, San Diego, CA, USA). Raw sequencing reads were filtered by removing adaptor sequences and low-quality reads to obtain clean reads. Clean reads were aligned to the assembled reference transcriptome, and gene expression levels were quantified as fragments per kilobase of transcript per million mapped reads (FPKM). Differentially expressed genes (*DEGs*) were identified using DESeq2 based on |log_2_(fold change)| ≥ 1 and adjusted *p*-value < 0.05. Functional annotation and enrichment analyses were performed using Gene Ontology (GO) and Kyoto Encyclopedia of Genes and Genomes (KEGG) pathway analyses. Because high-quality reference genomes are currently unavailable for *Kobresia tibetica* and *Blysmus sinocompressus*, a de novo transcriptome assembly strategy was applied. Clean reads from all samples were assembled using Trinity. The assembled transcripts were optimized, and the longest transcript within each transcript cluster was selected as a unigene. These unigenes were used as reference sequences for subsequent gene expression quantification, differential expression analysis, and functional annotation. Clean reads from each sample were aligned to the Trinity-assembled transcriptome to obtain expression profiles.

#### 4.4.2. qRT-PCR Validation of Candidate Genes

Based on transcriptomic analysis and previous studies, eight waterlogging-responsive candidate genes were selected for qRT-PCR validation. These genes included hypoxia-responsive and anaerobic metabolism-related genes (*ADH*, *PDC*, and *HRE*), antioxidant defense-related genes (*SOD*, *CAT*, and *APX*), a carbohydrate metabolism-related gene (*SUS*), and a stress signaling-related gene (*ERF*). Root samples from the CK, SW, MSW, and SSW treatments were used for qRT-PCR analysis to validate the expression patterns of key candidate genes under different waterlogging intensities. Total RNA was reverse-transcribed into cDNA, which was used as the template for qRT-PCR analysis. The *β-actin* gene was used as the internal reference gene, and relative gene expression levels were calculated using the 2^−ΔΔCt^ method. The specificity of amplification was confirmed by melting curve analysis, and the amplification efficiencies of all primer pairs ranged from 96% to 104% (Table 2). Each biological replicate included three technical replicates. Primer sequences, amplicon sizes, and amplification efficiencies are provided in Table 2.

### 4.5. Statistical Analysis

Statistical analyses were performed using SPSS Statistics version 26.0 (IBM Corp., Armonk, NY, USA). Differences among treatments were analyzed using one-way analysis of variance (ANOVA), followed by Duncan’s multiple range test at *p* < 0.05. Pearson correlation analysis and principal component analysis (PCA) were conducted to evaluate the relationships among physiological traits, root characteristics, and biochemical parameters

### 4.6. Transcriptome Data Analysis

Differentially expressed genes (*DEGs*) were identified based on transcriptome sequencing data. Gene Ontology (GO) enrichment analysis and Kyoto Encyclopedia of Genes and Genomes (KEGG) pathway enrichment analysis were performed to identify the functional categories and metabolic pathways associated with DEGs.

## 5. Conclusions

This study demonstrated that *Kobresia tibetica* and *Blysmus sinocompressus* exhibited distinct adaptive strategies under waterlogging stress due to their different hydrological niches. *K. tibetica* maintained relatively stable root traits and physiological homeostasis under moderate waterlogging, whereas *B. sinocompressus* showed stronger activation of hypoxia-responsive pathways, antioxidant defense, and carbohydrate metabolism under severe waterlogging conditions. Transcriptomic analysis further revealed species-specific regulatory strategies associated with oxygen deficiency response, energy metabolism, and stress adaptation. These findings highlight the importance of integrating root functional traits, rhizosphere processes, and molecular regulation for understanding waterlogging adaptation of alpine wetland plants. However, this study was conducted under controlled experimental conditions, and further field observations and functional validation of key candidate genes are needed to better elucidate the long-term adaptation mechanisms of alpine wetland plants under changing hydrological environments.

## Figures and Tables

**Table 1:** Summary of RNA-seq sequencing quality of root samples from *Kobresia tibetica* and *Blysmus sinocompressus* under CK and MSW treatments.

Sample	Raw Reads	Raw Bases	Clean Reads	Clean Bases	Error Rate (%)	Q20 (%)	Q30 (%)	GC Content (%)
*K. tibetica*_CK1	53,082,404	8,015,443,004	52,631,752	7,794,001,108	0.0278	95.85	93.56	43.97
*K. tibetica*_CK2	41,562,184	6,275,889,784	41,202,700	6,107,067,933	0.0282	95.64	93.23	43.55
*K. tibetica*_CK3	61,250,986	9,248,898,886	60,632,432	9,048,952,958	0.0272	96.24	93.85	43.65
*K. tibetica*_MSW1	42,811,646	6,464,558,546	42,470,114	6,330,679,664	0.0271	96.22	93.97	43.36
*K. tibetica*_MSW2	48,720,628	7,356,814,828	48,362,440	7,186,943,339	0.027	96.28	94.07	42.99
*K. tibetica*_MSW3	43,861,958	6,623,155,658	43,481,750	6,463,189,905	0.0275	96.07	93.7	43.45
*B. sinocompressus*_CK1	48,531,780	7,328,298,780	48,028,902	6,985,413,007	0.0278	95.92	93.57	52.34
*B. sinocompressus*_CK2	41,093,996	6,205,193,396	40,700,306	6,055,607,812	0.0284	95.6	93.12	51.96
*B. sinocompressus*_CK3	42,067,378	6,352,174,078	41,629,742	6,184,096,065	0.0286	95.48	92.95	52.18
*B. sinocompressus*_MSW1	44,621,608	6,737,862,808	44,122,746	6,546,253,218	0.0281	95.76	93.27	52.49
*B. sinocompressus*_MSW2	44,090,920	6,657,728,920	43,636,276	6,481,182,066	0.0283	95.63	93.15	52.64
*B. sinocompressus*_MSW3	43,767,134	6,608,837,234	43,333,754	6,434,197,006	0.0281	95.73	93.28	52.14

**Table 2:** Primer sequences, amplicon sizes, and amplification efficiencies used for qRT-PCR analysis.

Gene	Function	Forward Primer (5′–3′)	Reverse Primer (5′–3′)	Amplicon Size (bp)	Efficiency (%)
*ADH2*	Alcohol dehydrogenase 2	CACTGCCTCAACTGAGTAAAC	CAGCAGCTTTGCAACGAA	150	96
*PDC*	Pyruvate decarboxylase	GAAGCAATTGACACAGCAACA	CCGTACACACAAGCAGCAGTA	160	101
*HRE*	Hypoxia-responsive *ERF*	GAAGCGTAAACCCGTCTCAGT	TTTGCTCGGGCACGAATCT	123	97
*SOD*	Cu/Zn-superoxide dismutase	GGGTTCCACGTCCATCAGTA	AGTCACATTGCCCAGGTCTC	180	102
*CAT*	Catalase	GGTGTTCACACCTTCACTCT	GAGATCCTGAGTAGCATGACTG	170	104
*APX*	Ascorbate peroxidase	CTGCCGTCCCCTTCCACCCA	CCGCCAGAGAGGGCAACAAT	154	96
*SUS*	Sucrose synthase	GGCTGTTGCCTGATGCTGTT	TGCTCGGTTCCAATGACCTT	140	96
*ERF*	Ethylene response factor	TCCATCGCCTGATCCCTTTG	GAAGCAAGCAAACCAAGCCA	167	98
*β-actin*	Internal reference gene	TGCTAGACTCGGGAGATGGTG	AAGTCAAGACGTAGGATTGCA	85	96

## Data Availability

The datasets generated during the current study are available from the corresponding author upon reasonable request. The transcriptome sequencing data generated in this study have been deposited in the NCBI Sequence Read Archive (SRA) database.
